# Effects of cerebellar transcranial direct current stimulation on reversal learning performance during threat of shock

**DOI:** 10.1016/j.ijchp.2025.100558

**Published:** 2025-03-20

**Authors:** Eline S. Kruithof, Yvette Witteveen, Eleni Kalligeri Skentzou, Maria-Eleni Theodorakopoulou, Jana Klaus, Dennis J.L.G. Schutter

**Affiliations:** Department of Experimental Psychology, Helmholtz Institute, Utrecht University, Heidelberglaan 1, 3584 CS, Utrecht, the Netherlands

**Keywords:** Affective state-dependency, Anxiety, Cerebellum, Impulsivity, Reversal learning, Threat of shock, Transcranial direct current stimulation

## Abstract

According to the cerebellar lateralization hypothesis of motivational direction, relative left-to-right dominant posterior cerebellar activity is associated with avoidance motivation and anxiety, whereas relative right-to-left dominant posterior cerebellar activity is associated with approach motivation and anger. The present study tested this hypothesis in avoidance-related behavior during rule learning combined with threat of shock. In a randomized double-blind sham-controlled between-subjects design, ninety healthy right-handed adult volunteers received either active (*n* = 45) or sham (*n* = 45) cerebellar left anodal-right cathodal transcranial direct current stimulation (tDCS) to the posterior cerebellum. During tDCS participants performed a gambling task with two changing reward-punishment schedules (reversals) and were believed to think that based on performance they could receive mild electric shocks to the forearm. Self-reported measures of anxiety, anger and impulsivity were assessed to examine affective state- and trait-dependent effects of cerebellar tDCS on reversal learning performance. Results showed no main effect of cerebellar tDCS on reversal learning performance. However, higher levels of shock anxiety were associated with increased reversal learning rate after the first reversal in the active compared to sham tDCS condition. Additionally, higher scores on trait impulsivity were associated with reduced reversal learning rate after the second reversal in the active compared to sham tDCS condition. These findings provide support for the cerebellar lateralization hypothesis of motivational direction and yield further evidence for context-relevant affective state- and trait-dependency in tDCS-related effects.

## Introduction

According to the cerebellar lateralization hypothesis of motivational direction, relative left-to-right dominant posterior cerebellar activity is associated with avoidance motivation and anxiety, whereas relative right-to-left dominant posterior cerebellar activity is associated with approach motivation and anger (Kruithof et al., 2022, 2024; Schutter, 2020). Avoidance motivation is directed away from a stimulus (Feltman & Elliot, 2012) and is associated with anxious avoidance-related behaviors in the face of threat (Corr, 2013). Conversely, approach motivation is directed towards appetitive and potentially rewarding stimuli (Feltman & Elliot, 2012; Harmon-Jones et al., 2013). Approach motivation is associated with the emotion anger and behaviors such as impulsivity and aggression (Kruithof et al., 2022). The cerebellar lateralization hypothesis of motivational direction stems from the well-documented frontal cortical lateralization of motivational direction (for a review see Kelley et al., 2017) and the contralateral structural neuro-anatomical connections between the cerebellum and frontal cortex (Palesi et al., 2015, 2017). Moreover, the cerebellar lateralization hypothesis is substantiated by empirical evidence of crossed hemispheric functions of the cerebellum compared to the frontal cortex (Wang et al., 2013).

Recently, we provided the first empirical evidence for cerebellar lateralization of motivational direction in approach-related behavior (Kruithof et al., 2024). In a double-blind sham-controlled within-subjects study, healthy volunteers received left cathodal-right anodal cerebellar tDCS to induce relative right-to-left dominant activity while engaging in a provocation task. Left cathodal-right anodal tDCS facilitated aggression in individuals with higher levels of state anger compared to sham tDCS, which was interpreted as a relative increase in approach motivation associated with relative right-to-left dominant cerebellar activity (Kruithof et al., 2024). This result concurs with the finding of a previous sham-controlled between-subjects study in healthy volunteers which reported a positive association between state anger and aggressive behavior after left anodal-right cathodal tDCS to the frontal cortex (Hortensius et al., 2012). By contrast, in another sham-controlled between-subjects study in healthy volunteers, left cathodal-right anodal frontal tDCS increased rumination after interpersonal insult (Kelley et al., 2013). This observation can be interpreted as a relative lack of approach-related behavior (Kelley et al., 2013). While these findings highlight state anger-dependent tDCS effects, evidence also supports anxiety-related state-dependency in non-invasive brain stimulation effects (Schutter et al., 2023).

The aim of the present double-blind sham-controlled between-subjects study was to further test the cerebellar lateralization hypothesis of motivational direction in avoidance-related behavior. To this end, left anodal-right cathodal tDCS or sham tDCS was applied to the posterior cerebellum of healthy volunteers during a reversal learning task combined with threat of shock to promote avoidance behavior (Pittig and Scherbaum, 2020; Paret & Bublatzky, 2020; Robinson et al., 2013). A reward- and punishment-based reversal learning task was used because it taps into the role of the cerebellum in minimizing action-related reward and punishment prediction errors through a predictive coding routine (Kruithof et al., 2023). Based on evidence that threat can promote reversal learning when relevant for threat avoidance (Robinson et al., 2013), we hypothesized that left anodal-right cathodal cerebellar tDCS would improve reversal learning performance during threat of shock. Additionally, we examined possible affective state- and trait-dependent effects of cerebellar tDCS by investigating interactions of cerebellar tDCS with self-reported measures of anxiety, anger and impulsivity on reversal learning performance. We anticipated an interaction of tDCS condition with self-reported anxiety measures on reversal learning performance.

## Methods

### Participants

Ninety healthy, right-handed, non-smoking adult volunteers (63 females, age range = 18–35) participated in the study in exchange for course credit or a monetary compensation. Participants reported no history of neurological or psychiatric conditions, family history of epilepsy, skin disease or allergy, heart disease, metal in the head, pacemaker or neurostimulator, pregnancy and use of medication (except for oral contraceptives). The required sample size was estimated by performing a power analysis in G*Power 3.1 (Faul et al., 2007) for a one-way ANOVA with the following settings: *f* = 0.3 (based on Wischnewski et al., 2020), α = 0.05, power = 0.8, number of groups = 2, and yielded a required sample size of 90 participants. All participants gave written informed consent. The study was approved by the ethics committee of the Faculty of Social and Behavioural Sciences of Utrecht University (protocol number: 23–0041).

### Reversal learning gambling task

A modified version of the reversal learning gambling task was used to assess reward- and punishment-based reversal learning (Kruithof et al., 2024; Wischnewski et al., 2016, 2020; Schutte et al., 2017). On each trial, participants chose one of two numerical values representing fictitious money that could be won or lost. Participants could choose the higher numerical value (i.e., relatively large reward/loss for correct/incorrect decision) or the lower numerical value (i.e., relatively small reward/loss for correct/incorrect decision). After the participant's decision feedback was provided and a score update was shown on the computer screen. The task consisted of three phases that differed in their reward-punishment contingency schedule. In phase 1 (blocks 1 and 2, trials 1–40), choosing the higher numerical value was rewarded in 70 % of the trials. In phase 2 (blocks 3 and 4, trials 41–80), the reward-punishment contingency was reversed, such that choosing the higher numerical value was rewarded in only 30 % of the trials. In phase 3 (blocks 5 and 6, trials 81–120), the reward-punishment contingency was again reversed, such that choosing the higher numerical value was rewarded in 70 % of the trials. Thus, in the first and third task phase choosing the higher numerical value was the optimal strategy, whereas choosing the lower numerical value was the optimal strategy in the second task phase. Instead of the 80–20 version that was used in previous studies (Kruithof et al., 2024; Wischnewski et al., 2016, 2020; Schutte et al., 2017), we currently used a task version with 70–30 and 30–70 reward-punishment contingencies to raise task difficulty and increase the interindividual range in reversal learning performance.

### Transcranial direct current stimulation (tDCS)

Bipolar transcranial electric stimulation was applied via two rubber electrodes (5 × 5 cm) placed in sponges covered with conductive gel using a battery-driven DC stimulator (CE 0118; NeuroConn GmbH, Ilmenau, Germany). The electrode sponges were placed under a lycra EEG cap for appropriate localization and fixation. The anodal electrode was positioned over the left cerebellum corresponding to electrode position P9 and the cathodal electrode was positioned over the right cerebellum corresponding to electrode position P10 of the International 10–10 EEG system. Active cerebellar tDCS was delivered for 15 min during the reversal learning gambling task at a current intensity of 2 mA (current density: 0.08 mA/cm^2^, total charge density: 72 mC/cm^2^) with a 30 s ramp-up and ramp-down phase at the start and end of the stimulation period, respectively. In the sham cerebellar tDCS condition, participants received active stimulation for 30 s after an initial 5 s ramp-up, followed by a ramp-down of 5 s. The impedance of the electrodes was kept below 10 kΩ throughout stimulation.

### Threat of shock

Electric shocks were delivered through two disk electrodes on the inside of the participant's left forearm using a Digitimer DS7A constant current stimulator (Digitimer Ltd., Welwyn Garden City, UK) and consisted of 625-ms trains of 2-ms pulses delivered at 200 Hz. The intensity of the shocks was determined individually in a shock work-up procedure (Baas, 2013; Baas et al., 2014). The work-up consisted of two to eight sample shocks that participants rated on a five-point scale ranging from ‘not annoying’ to ‘very annoying’. The intensity that participants rated as 4, corresponding to ‘quite annoying’, was the intensity used during the study.

### Affective state-trait factors

The State-Trait Anxiety Inventory (STAI; Spielberger et al., 1970) was used to assess state and trait anxiety. Both the state and trait anxiety scale consist of twenty items to which participants responded on a four-point scale ranging from 1 (not at all) to 4 (very much so). The state and trait anxiety scale have good reliability in non-clinical populations with a Cronbach's α of 0.97 and 0.95, respectively (Ortuño-Sierra et al., 2016). Shock anxiety was assessed with a single question asking the participant to indicate how anxious they felt about receiving electric shocks on a ten-point scale ranging from 0 (not at all) to 9 (very much so). Due to an experimenter error, anxiety for shocks was assessed on an eleven-point scale (0–10) for one third of the participants. These scores were rescaled to a ten-point scale. The state anger scale of the State-Trait Anger Expression Inventory-2 (STAXI-2; Spielberger, 1999) was used to assess state anger based on ten items to which participants responded on a four-point scale ranging from 1 (not at all) to 4 (very much so). The state anger scale has good reliability (Cronbach's α = 0.96) in non-clinical populations (Lievaart et al., 2016). The Barratt Impulsiveness Scale (BIS-11; Patton et al., 1995) assessed trait impulsivity based on 30 items to which participants responded on a four-point scale ranging from 1 (rarely/never) to 4 (almost always/always). The BIS-11 has good reliability (Cronbach's α = 0.83) in non-clinical populations (Stanford et al., 2009).

### Procedure

Volunteers were recruited via the university's online participant recruitment system and flyer advertising on campus. After signing up for the study, participants received the study information letter via e-mail. Participants were told that the aim of the study was to investigate the role of the cerebellum in decision-making during threat. They were invited to the lab once for approximately one hour. Participants were requested to refrain from caffeine-containing drinks and chocolate two hours before the test session and to refrain from alcohol 24 h in advance. At the start of the session, participants provided informed consent and filled out the screening form to check for contraindications of tDCS. Next, volunteers filled out the state anxiety and state anger scale. The disk electrodes were applied on the left forearm for the electric shock delivery, followed by the shock work-up procedure. Before the shock work-up, participants indicated how anxious they felt about receiving shocks, which was expected to reflect avoidance-related motivation for the shocks (Robinson et al., 2013). Next, participants were prepared for cerebellar tDCS and stimulation was started. Participants received either active or sham cerebellar tDCS and allocation to stimulation condition was random and counterbalanced. The experimenter entered a pre-assigned five-digit code into the DC stimulator that unbeknownst to the experimenter and the participant would initiate active or sham cerebellar tDCS. One and a half minutes after cerebellar tDCS was initiated, participants started with the task while receiving electric shocks to the forearm. Participants were told that they could receive shocks during the task and that the probability of receiving a shock depended on their performance on the task. In reality, all participants received three shocks during the task, one in each task phase, either the first, second, or third time they made an incorrect decision. After the task, participants filled out the trait anxiety scale and the BIS-11. At the end of the session, a transcranial electrical stimulation (tES) sensation questionnaire was administered on which the intensity of perceived sensations of tDCS were reported on a five-point scale ranging from 1 (none) to 5 (unbearable). Additionally, the onset and offset of perceived sensations were assessed on a three-point scale (1 = at the beginning of the stimulation, 2 = during the stimulation, 3 = towards the end of stimulation). The sensations itch, pain, burning sensation, heat under the electrodes, iron taste, fatigue, headache, neck pain, phosphenes, dizziness, nausea and concentration problems were assessed. Finally, participants were asked to indicate whether they thought they had received active or sham tDCS, allowing us to check whether blinding was successful, and were debriefed.

### Data reduction and statistical analysis

For the questionnaires, after reverse-scoring the relevant items, total scores were calculated by summing the scores of the items.

To test the effect of tDCS condition and interactions with the affective states and traits on reversal learning performance, mixed logit regression models were fitted separately for each task phase (i.e., before the first reversal, after the first reversal, and after the second reversal) with the binomial response (0 = lower numerical value decision, 1 = higher numerical value decision) as dependent variable. Models that included the two-way interaction between the fixed effects tDCS condition and trial number (1–20) tested the main effect of tDCS condition on reversal learning performance and the effect of tDCS condition on learning rate (i.e., the interaction between tDCS and trial number). Interactions of tDCS condition and trial number with affective states/traits were tested in models with a three-way interaction that included the affective state/trait score as additional fixed effect (i.e., both as a main effect and part of higher-order interactions). Models included a by-participant intercept and a by-participant slope for the effect of trial number, or, if the model did not converge or was overfitted (i.e., as indicated by a singular fit), a by-participant intercept only. All affective state/trait scores were *z*-transformed. Self-reported shock anxiety scores were missing for three participants. These participants were excluded from the models that tested the interaction with shock anxiety but were included in the other models. The individual *p*-values of all computed models were FDR-corrected across the *p*-values from all models to account for multiple testing.

To test whether the task with the 70–30 contingency ratio was effective at assessing reversal learning performance across tDCS conditions, a mixed logit regression model was fitted with the binomial response (0 = lower numerical value decision, 1 = higher numerical value decision) as dependent variable. The model included the interaction between the fixed effects block number (1–6) and trial number (1–20) as well as random intercepts for participants. Learning throughout the task was assessed by comparing the probability of a higher numerical value decision between consecutive task blocks using the *emmeans* function of the *emmeans* package in R (Lenth, 2024).

Mixed logit regression models were fitted with a binomial distribution and logit link function using the *glmer* function of the *lme4* package in R (Bates et al., 2015). Multicollinearity as indexed by a variance inflation factor ≥ 5, over- and underdispersion and outliers based on Cook's distance were evaluated for all models with the *check_collinearity, check_overdispersion* and *check_outliers* functions of the *performance* package (Lüdecke et al., 2021). For all models, absence of multicollinearity as well as over- and underdispersion was confirmed. The model that tested the main effect of tDCS condition on reversal learning performance in the second task phase contained four outliers. The participants to whom these numbers refer to were excluded from this model. No outliers were detected in the other models.

Separate independent samples *t*-tests were performed to assess differences between tDCS conditions in state and trait anxiety, shock anxiety, state anger, trait impulsivity and shock intensity. Additionally, separate independent samples *t*-tests were performed to assess differences between tDCS conditions in self-reported sensations during stimulation. For six participants, tES sensation data were incomplete, because responses were not registered (*n* = 1) or participants did not answer all the questions (*n* = 5). The partially available data of the five participants were included in the sensation analyses.

All statistical analyses were performed in R version 4.4.1 (R Core Team, 2024). The alpha level of significance was set to 0.05 (two-tailed) throughout.

## Results

Cerebellar tDCS was well tolerated and no adverse events were observed (see **Supplementary Table 4** for details). The average (*SD*) shock intensity was 2.21 (0.98) mA. No difference between tDCS conditions was observed in self-reported state anxiety (*t*(88) = −0.026, *p* = 0.979), trait anxiety (*t*(88) = −0.744, *p* = 0.459), shock anxiety (*t*(85) = 0.129, *p* = 0.897), state anger (*t*(88) = −0.815, *p* = 0.417), trait impulsivity (*t*(88) = −0.380, *p* = 0.705) and shock intensity (*t*(87) = −0.538, *p* = 0.592). Table 1 displays the descriptives of the sample demographics and self-reported affective state-trait scores per tDCS condition.

**Table 1:** Descriptive statistics of the sample demographics and self-reported affective state-trait scores per tDCS condition (presented as mean ± standard deviation for continuous variables, *n* = 45 per tDCS condition unless indicated otherwise).

	Sham tDCS	Active tDCS
Demographics		
Age	23.81 ± 2.87 (*n* = 43)	24.31 ± 3.20
Number of females	32	31
		
Affective states and traits		
Baseline state anxiety (range: 20–80)	35.96 ± 7.38	36 ± 8.64
Trait anxiety (range: 20–80)	41.24 ± 9.48	42.87 ± 11.14
Shock anxiety (range: 0–9)	4.26 ± 2.01 (*n* = 43)	4.20 ± 2.34 (*n* = 44)
Baseline state anger (range: 10–40)	11 ± 1.76	11.44 ± 3.21
Trait impulsivity (range: 30–120)	59.76 ± 7.35	60.38 ± 8.18

**Fig. 1:**
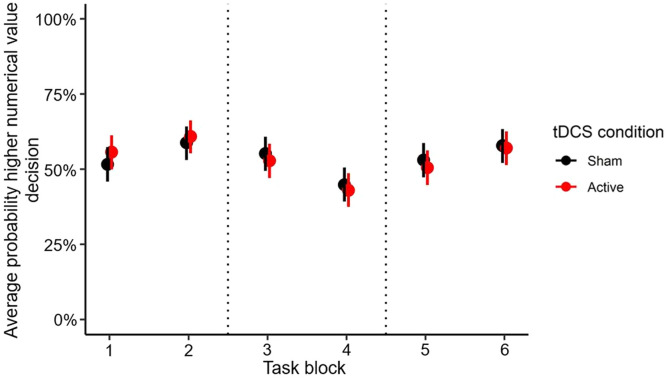
Average probability of a higher numerical value decision per task block for each tDCS condition. Error bars indicate standard errors of the mean. Dotted lines indicate the first reversal (from block 2 to block 3; from the higher towards the lower numerical value as optimal strategy) and the second reversal (from block 4 to block 5; from the lower towards the higher numerical value as optimal strategy).

The probability of a higher numerical value decision differed significantly between consecutive task blocks across tDCS conditions (*p*s ≤ 0.014) in line with the underlying reward-punishment contingency (Fig. 1).

For the first task phase, no main effect of tDCS condition on the probability higher numerical value decisions (*odds ratio* = 0.94, *SE* = 0.08, *z* = −0.74, *p* = 0.907) was observed, nor did tDCS condition affect learning rate (*odds ratio* = 1.00, *SE* = 0.00, *z* = 0.13, *p* = 0.969). A main effect of trial number was found (*odds ratio* = 1.01, *SE* = 0.00, *z* = 4.43, *p* < 0.001), indicating that the probability of a higher numerical value decision increased across trials, in line with the reward-punishment contingency. For all mixed logit regression models that included a self-reported affective state/trait, a main effect of trial number was found as well (*p*s < 0.001). No other effects were statistically significant (*p*s ≥ 0.387; **Supplementary Table 1A-1F**). It should be noted that the model that tested interactions with impulsivity did not converge with either the maximal or reduced random effects structure. Therefore, these results, based on the reduced random effects structure, should be interpreted with caution.

**Fig. 2:**
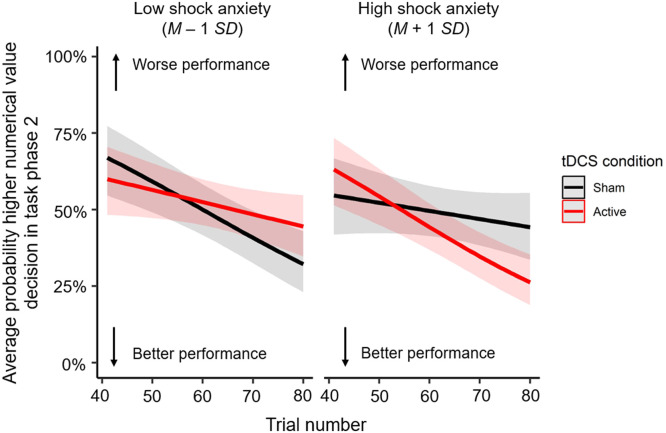
Interaction between tDCS condition, trial number and shock anxiety on the average probability of a higher numerical value decision in task phase 2 (i.e., after the first reversal at trial 41, when choosing the lower numerical value is the optimal strategy). The interaction is illustrated for low (M – 1 *SD*; 2.06) and high (M + 1 *SD*; 6.40) levels of shock anxiety.

For the second task phase, no main effect of tDCS condition on the probability higher numerical value decisions (*odds ratio* = 0.99, *SE* = 0.10, *z* = −0.07, *p* = 0.969) was observed, nor did tDCS condition affect learning rate (*odds ratio* = 1.00, *SE* = 0.00, *z* = 0.56, *p* = 0.969). A main effect of trial number was found (*odds ratio* = 0.98, *SE* = 0.00, *z* = −7.45, *p* < 0.001), indicating that the probability of a higher numerical value decision decreased across trials, in line with the reward-punishment contingency. For all mixed logit regression models that included a self-reported affective state/trait, a main effect of trial number was found as well (*p*s < 0.001). Additionally, a significant three-way interaction between tDCS condition, trial number and shock anxiety predicted the probability of a higher numerical value decision after the first reversal in task phase 2 (*odds ratio* = 1.01, *SE* = 0.00, *z* = 2.81, *p* = 0.025; Fig. 2). Active tDCS reduced learning rate in individuals with lower levels of shock anxiety compared to sham tDCS. In contrast, active tDCS increased learning rate in individuals with higher levels of shock anxiety compared to sham tDCS. No other effects were statistically significant (*p*s ≥ 0.490; **Supplementary Table 2A-2F**).

**Fig. 3:**
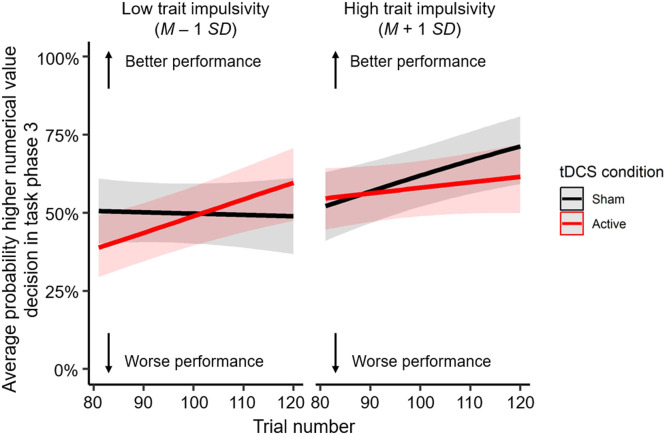
Interaction between tDCS condition, trial number and trait impulsivity score on the average probability of a higher numerical value decision in task phase 3 (i.e., after the second reversal at trial 81, when choosing the higher numerical value is the optimal strategy). The interaction is illustrated for low (M – 1 *SD*; 52.38) and high (M + 1 *SD*; 67.76) trait impulsivity scores.

For the third task phase, no main effect of tDCS condition on the probability higher numerical value decisions (*odds ratio* = 1.10, *SE* = 0.12, *z* = 0.83, *p* = 0.907) was observed, nor did tDCS condition affect learning rate (*odds ratio* = 1.00, *SE* = 0.00, *z* = −0.80, *p* = 0.907). A main effect of trial number was found (*odds ratio* = 1.01, *SE* = 0.00, *z* = 3.34, *p* = 0.006), indicating that the probability of a higher numerical value decision increased across trials, in line with the reward-punishment contingency. For all mixed logit regression models that included a self-reported affective state/trait, a main effect of trial number was found as well (*p*s ≤ 0.006). Additionally, a significant three-way interaction between tDCS condition, trial number and trait impulsivity score predicted the probability of a higher numerical value decision after the second reversal in task phase 3 (*odds ratio* = 1.01, *SE* = 0.00, *z* = 2.80, *p* = 0.025; Fig. 3). Active tDCS increased learning rate in individuals with lower levels of trait impulsivity compared to sham tDCS. In contrast, active tDCS reduced learning rate in individuals with higher levels of trait impulsivity compared to sham tDCS. No other effects were statistically significant (*p*s ≥ 0.083; **Supplementary Table 3A-3F**).

A trend of a higher level of self-reported dizziness (*t*(83) = −1.792, *p* = 0.077) and fatigue (*t*(84) = −1.680, *p* = 0.097) in the active as compared to the sham tDCS condition was found (**Supplementary Table 4**). The other sensations did not differ between tDCS conditions (*p*s ≥ 0.123; **Supplementary Table 4**). Thirty six out of 89 participants (41 %) guessed their stimulation condition correctly, indicating that blinding was successful on the group level (*X*^2^_1_ (89) = 3.247, *p* = 0.072).

## Discussion

This study examined the cerebellar lateralization hypothesis of motivational direction in avoidance-related behavior during a reversal learning task combined with threat of shock. Contrary to our hypothesis, cerebellar tDCS did not affect reversal learning performance. This finding is a replication of our previous study on the cerebellar lateralization of motivational direction where no main effect of tDCS on approach-related behavior was found using the reversed cerebellar bipolar tDCS montage (i.e., left cathodal-right anodal; Kruithof et al., 2024). In addition, we replicated our previous finding of an affective state-dependent effect of cerebellar tDCS. That is, the present results show a shock anxiety-dependent effect of cerebellar tDCS on reversal learning rate after the first reversal. Furthermore, a trait impulsivity-dependent effect of cerebellar tDCS on reversal learning rate after the second reversal was found.

Active tDCS reduced learning rate after the first reversal compared to sham tDCS in individuals with lower levels of shock anxiety but increased learning rate in individuals with higher levels of shock anxiety. Individuals who reported more anxiety for electric shocks and underwent active tDCS were arguably more motivated to learn in order to avoid the electric shocks (Robinson et al., 2013). This is in agreement with the idea of a link between relative left-to-right dominant cerebellar activity and avoidance motivation. By contrast, in the sham tDCS condition, a higher level of shock anxiety had no beneficial effect on learning. Likewise, active tDCS did not facilitate learning when shock anxiety, and presumably the motivation to avoid shocks, was relatively low. Thus, levels of shock anxiety and avoidance motivation may have been suboptimal in the latter instances (Salehi et al., 2010) to facilitate learning. The shock anxiety-dependent effect of cerebellar tDCS was specific for learning after the first, but not second reversal. This finding is in line with the well-documented association between anxiety and risk avoidance (Lorian and Grisham, 2011; Maner & Schmidt, 2006; Maner et al., 2007). Higher shock anxiety in combination with active cerebellar tDCS arguably facilitated the preference for the lower (low-risk) numerical value. That is, choosing the lower numerical value yielded a higher probability of incurring a relatively small loss in case of an incorrect choice compared to the higher numerical value. Shock anxiety was significantly correlated with state anxiety (*r* = 0.32, *p* = 0.003), but not with trait anxiety (*r* = 0.18, *p* = 0.098) and state anger (*r* = 0.04, *p* = 0.746). While significantly related to state anxiety, shock anxiety may have captured the behavioral component of the task in a threat context more adequately. The close link between affective state and context (Robinson et al., 2013) may explain why, apart from shock anxiety, no other interactions of cerebellar tDCS with self-reported anxiety and anger measures on reversal learning performance were found in the present study. It should be noted that the shock anxiety-dependent *increase* in learning rate during active tDCS under threat of shock may seem paradoxical to the previously reported evidence that threat of shock can impair reversal learning (Paret & Bublatzky, 2020). Importantly, in contrast to the present study, participants in the study of Paret and Bublatzky (2020) were told that probability of receiving shocks during the reversal learning task did not depend on their task performance. Arguably, the subjectively experienced feeling of control on shock avoidance may be a relevant factor underlying the direction of the effect, such that a lack of control interferes with reversal learning, whereas a feeling of control facilitates reversal learning (Robinson et al., 2013).

Active tDCS increased learning rate after the second reversal compared to sham tDCS in individuals with lower levels of impulsivity but reduced learning rate in individuals with higher levels of impulsivity. In the sham tDCS condition, impulsivity was positively associated with reversal learning performance. Impulsivity can be a useful trait when reward sensitivity and/or punishment insensitivity are beneficial for task performance (Smillie & Jackson, 2006). After the second reversal, choosing the higher (relatively more rewarding in case of a correct choice) numerical value is the optimal strategy. While this strategy is congruent with the approach-related reward-seeking tendency of more impulsive individuals (Elliott et al., 2023; Moeller et al., 2001), the opposite (i.e., relatively reward-insensitive/punishment-sensitive) likely holds for individuals scoring lower on impulsivity. For lower levels of impulsivity, active tDCS improved learning rate after the second reversal compared to sham tDCS. Mild levels of avoidance-related anxiety (Sandi, 2013) can promote reversal learning when relevant for threat avoidance (Robinson et al., 2013), supporting the idea that left anodal-right cathodal cerebellar tDCS may have contributed to a motivational stance of avoidance. For higher levels of impulsivity, worse learning during active compared to sham tDCS may suggest that approach-related impulsivity overruled the putative effect of shock anxiety and avoidance motivation during active tDCS (Corr, 2002). The trait impulsivity-dependent effect of cerebellar tDCS was specific for learning after the second reversal, which may be explained by habituation to the electric shocks as the task progressed. Speculatively, in the first part of the task, the influence of shock anxiety may have been more dominant, possibly overruling a trait impulsivity-dependent effect of tDCS.

In addition, we examined post-hoc whether the shock anxiety- and trait impulsivity-dependent effects of tDCS condition on learning rate could be explained by differences in experienced dizziness and fatigue. To this end, dizziness and fatigue were separately added as a main fixed effect in the mixed logit regression models. After controlling for dizziness and fatigue, the shock anxiety- and impulsivity-dependent effects of tDCS condition remained significant, indicating that these effects were not driven by higher perceived dizziness and fatigue under active tDCS (see **Supplementary Table 5A-D** for details).

Recently, we showed that left cathodal-right anodal cerebellar tDCS in combination with higher levels of state anger facilitated approach-related behavior (Kruithof et al., 2024). The current findings provide further support for the cerebellar lateralization hypothesis of motivational direction, where relative left-to-right and right-to-left dominant cerebellar activity are associated with avoidance and approach motivation, respectively. This cerebellar lateralization of motivational direction is reversed compared to the frontal cortex (Kelley et al., 2017). Given the contralateral structural and functional connections between the cerebellum and frontal cortex (Palesi et al., 2015, 2017; Wang et al., 2013), findings can be understood in terms of cerebello-cortical interactions. The role of cerebello-cortical interactions in approach- and avoidance-related behavior warrants further research. For example, the local effect of a tDCS-induced cerebellar lateralization on distal cortical lateralization could be tested and related to frontal electrophysiological and behavioral indices of approach- and avoidance-related behavior (Kelley et al., 2017). Additionally, it would be interesting to induce transient lateralization with tDCS in the cerebellum and frontal cortex simultaneously, either congruent (e.g., cerebellum left anodal-right cathodal and frontal left cathodal-right anodal) or incongruent with contralateral cerebellar-cortical connections, and test the effects on approach- and avoidance-related behavior.

Our findings may contribute to potential treatments for mood disorders characterized by imbalances in avoidance and approach motivation. For example, the cerebellum as opposed to the prefrontal cortex (Perera et al., 2016; Schutter, 2009) may be a potential target location for non-invasive brain stimulation in major depressive disorder (Schutter & van Honk, 2005). This is supported by observations of reductions in grey matter volume and density in individuals with major depressive disorder (Frodl et al., 2008; Peng et al., 2011). In particular, cerebellar non-invasive brain stimulation could be applied to reduce avoidance and increase approach motivation (Trew, 2011). Moreover, results suggest that the effects of tDCS can be state- and context-dependent and consequently can influence clinical outcome (Schutter et al., 2023).

Several limitations of the study should be mentioned. First, individual differences in neuroanatomy and -morphology likely contributed to interindividual differences in the focality of the applied tDCS montage (Maas et al., 2023). Simulations of electric fields based on individual structural MRI scans may broaden our understanding of how tDCS interacts with the cerebellum. Second, we did not test the effect of the reversed bipolar cerebellar tDCS montage (i.e., left cathodal-right anodal), so the specificity of the current findings for a relative left-to-right dominant cerebellar lateralization cannot be established.

In conclusion, the findings provide additional support for the cerebellar lateralization hypothesis of motivational direction and yield further evidence for context-relevant affective state- and trait-dependency in tDCS-related effects (Schutter et al., 2023).

## Declaration of competing interest

The authors declare that they have no known competing financial interests or personal relationships that could have appeared to influence the work reported in this paper.

## Appendix Supplementary materials

Image, application 1
