# Supplementary material for: Effects of cerebellar transcranial direct current stimulation on reversal learning performance during threat of shock
Source: Int J Clin Health Psychol. 2025 Mar 20;25(1):100558. doi: 10.1016/j.ijchp.2025.100558 (PMC11979461; doi:10.1016/j.ijchp.2025.100558)
Supplement: Supplementary file 1 [file mmc1.docx]

**Supplementary Materials**

**Table 1A.** Mixed effects logistic regression results predicting the probability of a higher numerical value decision in task phase 1 from tDCS condition and trial number (*n* = 90).

| *Predictors* | *Odds ratio* | *SE* | *z* | *p (corrected)* |
| --- | --- | --- | --- | --- |
| Intercept | 1.01 | 0.09 | 0.13 | 0.969 |
| tDCS | 0.94 | 0.08 | -0.74 | 0.907 |
| Trial | 1.01 | 0.00 | 4.43 | **<0.001** |
| tDCS * trial | 1.00 | 0.00 | 0.13 | 0.969 |
| Number of observations | | 3600  0.008 / 0.088 | |  |
| Marginal R^2^ / Conditional R^2^ | |  |  |  |
| SE = standard error of the mean, tDCS = transcranial direct current stimulation | | | | |

**Table 1B.** Mixed effects logistic regression results predicting the probability of a higher numerical value decision in task phase 1 from tDCS condition, trial number and state anxiety (*n* = 90).

| *Predictors* | *Odds ratio* | *SE* | *z* | *p (corrected)* |
| --- | --- | --- | --- | --- |
| Intercept | 1.01 | 0.09 | 0.12 | 0.969 |
| tDCS | 0.94 | 0.08 | -0.74 | 0.907 |
| Trial | 1.01 | 0.00 | 4.44 | **<0.001** |
| State anxiety | 1.06 | 0.10 | 0.63 | 0.945 |
| tDCS * trial | 1.00 | 0.00 | 0.11 | 0.969 |
| tDCS * state anxiety | 0.96 | 0.09 | -0.42 | 0.969 |
| Trial * state anxiety | 1.00 | 0.00 | -0.06 | 0.969 |
| tDCS * trial * state anxiety | 1.00 | 0.00 | -0.90 | 0.907 |
| Observations | | 3600 |  |  |
| Marginal R^2^ / Conditional R^2^ | | 0.012 / 0.088 |  |  |
| SE = standard error of the mean, tDCS = transcranial direct current stimulation | | | | |

**Table 1C.** Mixed effects logistic regression results predicting the probability of a higher numerical value decision in task phase 1 from tDCS condition, trial number and trait anxiety (*n* = 90).

| *Predictors* | *Odds ratio* | *SE* | *z* | *p (corrected)* |
| --- | --- | --- | --- | --- |
| Intercept | 1.01 | 0.09 | 0.09 | 0.969 |
| tDCS | 0.94 | 0.08 | -0.68 | 0.918 |
| Trial | 1.01 | 0.00 | 4.37 | **<0.001** |
| Trait anxiety | 1.08 | 0.10 | 0.81 | 0.907 |
| tDCS * trial | 1.00 | 0.00 | 0.10 | 0.969 |
| tDCS * trait anxiety | 0.96 | 0.09 | -0.43 | 0.969 |
| Trial * trait anxiety | 1.00 | 0.00 | -0.33 | 0.969 |
| tDCS * trial * trait anxiety | 1.00 | 0.00 | -0.73 | 0.907 |
| Observations |  | 3600 |  |  |
| Marginal R^2^ / Conditional R^2^ | | 0.011 / 0.089 | |  |
| SE = standard error of the mean, tDCS = transcranial direct current stimulation | | | | |

**Table 1D.** Mixed effects logistic regression results predicting the probability of a higher numerical value decision in task phase 1 from tDCS condition, trial number and shock anxiety (*n* = 87).

| *Predictors* | *Odds ratio* | *SE* | *z* | *p (corrected)* |
| --- | --- | --- | --- | --- |
| Intercept | 1.01 | 0.09 | 0.11 | 0.969 |
| tDCS | 0.93 | 0.09 | -0.84 | 0.907 |
| Trial | 1.01 | 0.00 | 4.42 | **<0.001** |
| Shock anxiety | 1.07 | 0.10 | 0.73 | 0.907 |
| tDCS * trial | 1.00 | 0.00 | 0.25 | 0.969 |
| tDCS * shock anxiety | 0.98 | 0.09 | -0.21 | 0.969 |
| Trial * shock anxiety | 0.99 | 0.00 | -1.74 | 0.387 |
| tDCS * trial * shock anxiety | 1.00 | 0.00 | -0.93 | 0.907 |
| Observations |  | 3480 |  |  |
| Marginal R^2^ / Conditional R^2^ | | 0.011 / 0.093 | |  |
| SE = standard error of the mean, tDCS = transcranial direct current stimulation | | | | |

**Table 1E.** Mixed effects logistic regression results predicting the probability of a higher numerical value decision in task phase 1 from tDCS condition, trial number and state anger (*n* = 90).

| *Predictors* | *Odds ratio* | *SE* | *z* | *p (corrected)* |
| --- | --- | --- | --- | --- |
| Intercept | 1.01 | 0.09 | 0.07 | 0.969 |
| tDCS | 0.94 | 0.08 | -0.71 | 0.907 |
| Trial | 1.01 | 0.00 | 4.38 | **<0.001** |
| State anger | 1.02 | 0.11 | 0.22 | 0.969 |
| tDCS * trial | 1.00 | 0.00 | 0.12 | 0.969 |
| tDCS * state anger | 0.94 | 0.10 | -0.54 | 0.969 |
| Trial * state anger | 1.00 | 0.00 | 0.02 | 0.987 |
| tDCS * trial * state anger | 1.00 | 0.00 | -0.35 | 0.969 |
| Observations |  | 3600 |  |  |
| Marginal R^2^ / Conditional R^2^ | | 0.010 / 0.088 | |  |
| SE = standard error of the mean, tDCS = transcranial direct current stimulation | | | | |

**Table 1F.** Mixed effects logistic regression results predicting the probability of a higher numerical value decision in task phase 1 from tDCS condition, trial number and trait impulsivity (*n* = 90).

| *Predictors* | *Odds ratio* | *SE* | *z* | *p (corrected)* |
| --- | --- | --- | --- | --- |
| Intercept | 1.01 | 0.09 | 0.14 | 0.969 |
| tDCS | 0.94 | 0.08 | -0.71 | 0.907 |
| Trial | 1.01 | 0.00 | 4.40 | **<0.001** |
| Trait impulsivity | 1.05 | 0.10 | 0.53 | 0.969 |
| tDCS * trial | 1.00 | 0.00 | 0.13 | 0.969 |
| tDCS * trait impulsivity | 1.03 | 0.09 | 0.29 | 0.969 |
| Trial * trait impulsivity | 1.00 | 0.00 | 0.02 | 0.987 |
| tDCS * trial * trait impulsivity | 1.00 | 0.00 | -0.56 | 0.969 |
| Observations  Marginal R^2^ / Conditional R^2^ | | 3600  0.008 / 0.088 | |  |
|  |  |  |  |  |
| SE = standard error of the mean, tDCS = transcranial direct current stimulation | | | | |

**Table 2A.** Mixed effects logistic regression results predicting the probability of a higher numerical value decision in task phase 2 from tDCS condition and trial number (*n* = 86).

| *Predictors* | *Odds ratio* | *SE* | *z* | *p (corrected)* |
| --- | --- | --- | --- | --- |
| Intercept | 1.49 | 0.15 | 3.95 | <0.001 |
| tDCS | 0.99 | 0.10 | -0.07 | 0.969 |
| Trial | 0.98 | 0.00 | -7.45 | **<0.001** |
| tDCS * trial | 1.00 | 0.00 | 0.56 | 0.969 |
| Observations  Marginal R^2^ / Conditional R^2^ | | 3440  0.020 / 0.129 | |  |
|  |  |  |  |  |
| SE = standard error of the mean, tDCS = transcranial direct current stimulation | | | | |

**Table 2B.** Mixed effects logistic regression results predicting the probability of a higher numerical value decision in task phase 2 from tDCS condition, trial number and state anxiety (*n* = 90).

| *Predictors* | *Odds ratio* | *SE* | *z* | *p (corrected)* |
| --- | --- | --- | --- | --- |
| Intercept | 1.61 | 0.21 | 3.57 | <0.001 |
| tDCS | 1.02 | 0.14 | 0.18 | 0.969 |
| Trial | 0.98 | 0.00 | -5.30 | **<0.001** |
| State anxiety | 0.99 | 0.13 | -0.08 | 0.969 |
| tDCS * trial | 1.00 | 0.00 | 0.22 | 0.969 |
| tDCS * state anxiety | 0.83 | 0.11 | -1.38 | 0.668 |
| Trial * state anxiety | 1.00 | 0.00 | 0.47 | 0.969 |
| tDCS * trial * state anxiety | 1.00 | 0.00 | 1.00 | 0.907 |
| Observations  Marginal R^2^ / Conditional R^2^ | | 3600  0.024 / 0.178 | |  |
|  |  |  |  |  |
| SE = standard error of the mean, tDCS = transcranial direct current stimulation | | | | |

**Table 2C.** Mixed effects logistic regression results predicting the probability of a higher numerical value decision in task phase 2 from tDCS condition, trial number and trait anxiety (*n* = 90).

| *Predictors* | *Odds ratio* | *SE* | *z* | *p (corrected)* |
| --- | --- | --- | --- | --- |
| Intercept | 1.59 | 0.21 | 3.46 | 0.006 |
| tDCS | 1.03 | 0.14 | 0.19 | 0.969 |
| Trial | 0.98 | 0.00 | -5.23 | **<0.001** |
| Trait anxiety | 1.03 | 0.14 | 0.21 | 0.969 |
| tDCS * trial | 1.00 | 0.00 | 0.20 | 0.969 |
| tDCS * trait anxiety | 0.88 | 0.12 | -0.97 | 0.907 |
| Trial * trait anxiety | 1.00 | 0.00 | -0.31 | 0.969 |
| tDCS * trial * trait anxiety | 1.00 | 0.00 | 0.42 | 0.969 |
| Observations  Marginal R^2^ / Conditional R^2^ | | 3600  0.023 / 0.178 | |  |
|  |  |  |  |  |
| SE = standard error of the mean, tDCS = transcranial direct current stimulation | | | | |

**Table 2D.** Mixed effects logistic regression results predicting the probability of a higher numerical value decision in task phase 2 from tDCS condition, trial number and shock anxiety (*n* = 87).

| *Predictors* | *Odds ratio* | *SE* | *z* | *p (corrected)* |
| --- | --- | --- | --- | --- |
| Intercept | 1.62 | 0.21 | 3.75 | <0.001 |
| tDCS | 0.99 | 0.13 | -0.11 | 0.969 |
| Trial | 0.97 | 0.00 | -5.82 | **<0.001** |
| Shock anxiety | 0.91 | 0.12 | -0.74 | 0.907 |
| tDCS * trial | 1.00 | 0.00 | 0.47 | 0.969 |
| tDCS * shock anxiety | 0.84 | 0.11 | -1.34 | 0.699 |
| Trial * shock anxiety | 1.00 | 0.00 | 0.13 | 0.969 |
| tDCS * trial * shock anxiety | 1.01 | 0.00 | 2.81 | **0.025** |
| Observations  Marginal R^2^ / Conditional R^2^ | | 3480  0.032 / 0.173 | |  |
|  |  |  |  |  |
| SE = standard error of the mean, tDCS = transcranial direct current stimulation | | | | |

**Table 2E.** Mixed effects logistic regression results predicting the probability of a higher numerical value decision in task phase 2 from tDCS condition, trial number and state anger (*n* = 90).

| *Predictors* | *Odds ratio* | *SE* | *z* | *p (corrected)* |
| --- | --- | --- | --- | --- |
| Intercept | 1.60 | 0.21 | 3.51 | <0.001 |
| tDCS | 1.03 | 0.14 | 0.25 | 0.969 |
| Trial | 0.98 | 0.00 | -5.35 | **<0.001** |
| State anger | 1.14 | 0.18 | 0.81 | 0.907 |
| tDCS * trial | 1.00 | 0.00 | 0.13 | 0.969 |
| tDCS * state anger | 0.94 | 0.15 | -0.37 | 0.969 |
| Trial * state anger | 0.99 | 0.01 | -1.02 | 0.907 |
| tDCS * trial * state anger | 1.00 | 0.01 | -0.71 | 0.907 |
| Observations  Marginal R^2^ / Conditional R^2^ | | 3600  0.027 / 0.178 | |  |
|  |  |  |  |  |
| SE = standard error of the mean, tDCS = transcranial direct current stimulation | | | | |

**Table 2F.** Mixed effects logistic regression results predicting the probability of a higher numerical value decision in task phase 2 from tDCS condition, trial number and trait impulsivity (*n* = 90).

| *Predictors* | *Odds ratio* | *SE* | *z* | *p (corrected)* |
| --- | --- | --- | --- | --- |
| Intercept | 1.61 | 0.21 | 3.57 | <0.001 |
| tDCS | 1.03 | 0.14 | 0.23 | 0.969 |
| Trial | 0.98 | 0.00 | -5.37 | **<0.001** |
| Trait impulsivity | 1.23 | 0.16 | 1.57 | 0.490 |
| tDCS * trial | 1.00 | 0.00 | 0.22 | 0.969 |
| tDCS * trait impulsivity | 0.96 | 0.13 | -0.27 | 0.969 |
| Trial * trait impulsivity | 1.00 | 0.00 | -0.67 | 0.919 |
| tDCS * trial * trait impulsivity | 0.99 | 0.00 | -1.11 | 0.907 |
| Observations  Marginal R^2^ / Conditional R^2^ | | 3600  0.034 / 0.178 | |  |
|  |  |  |  |  |
| SE = standard error of the mean, tDCS = transcranial direct current stimulation | | | | |

**Table 3A.** Mixed effects logistic regression results predicting the probability of a higher numerical value decision in task phase 3 from tDCS condition and trial number (*n* = 90).

| *Predictors* | *Odds ratio* | *SE* | *z* | *p (corrected)* |
| --- | --- | --- | --- | --- |
| Intercept | 0.96 | 0.11 | -0.38 | 0.969 |
| tDCS | 1.10 | 0.12 | 0.83 | 0.907 |
| Trial | 1.01 | 0.00 | 3.34 | **0.006** |
| tDCS * trial | 1.00 | 0.00 | -0.80 | 0.907 |
| Observations  Marginal R^2^ / Conditional R^2^ | | 3600  0.005 / 0.204 | |  |
|  |  |  |  |  |
| SE = standard error of the mean, tDCS = transcranial direct current stimulation | | | | |

**Table 3B.** Mixed effects logistic regression results predicting the probability of a higher numerical value decision in task phase 3 from tDCS condition, trial number and state anxiety (*n* = 90).

| *Predictors* | *Odds ratio* | *SE* | *z* | *p (corrected)* |
| --- | --- | --- | --- | --- |
| Intercept | 0.96 | 0.11 | -0.37 | 0.969 |
| tDCS | 1.10 | 0.12 | 0.83 | 0.907 |
| Trial | 1.01 | 0.00 | 3.33 | **0.006** |
| State anxiety | 1.07 | 0.12 | 0.57 | 0.969 |
| tDCS * trial | 1.00 | 0.00 | -0.79 | 0.907 |
| tDCS * state anxiety | 0.83 | 0.09 | -1.69 | 0.414 |
| Trial * state anxiety | 1.00 | 0.00 | -1.02 | 0.907 |
| tDCS * trial * state anxiety | 1.00 | 0.00 | 0.44 | 0.969 |
| Observations  Marginal R^2^ / Conditional R^2^ | | 3600  0.011 / 0.204 | |  |
|  |  |  |  |  |
| SE = standard error of the mean, tDCS = transcranial direct current stimulation | | | | |

**Table 3C.** Mixed effects logistic regression results predicting the probability of a higher numerical value decision in task phase 3 from tDCS condition, trial number and trait anxiety (*n* = 90).

| *Predictors* | *Odds ratio* | *SE* | *z* | *p (corrected)* |
| --- | --- | --- | --- | --- |
| Intercept | 0.95 | 0.11 | -0.44 | 0.969 |
| tDCS | 1.11 | 0.12 | 0.93 | 0.907 |
| Trial | 1.01 | 0.00 | 3.35 | **0.006** |
| Trait anxiety | 1.13 | 0.13 | 1.06 | 0.907 |
| tDCS * trial | 1.00 | 0.00 | -1.01 | 0.907 |
| tDCS * trait anxiety | 0.91 | 0.10 | -0.84 | 0.907 |
| Trial * trait anxiety | 0.99 | 0.00 | -2.39 | 0.083 |
| tDCS * trial * trait anxiety | 1.00 | 0.00 | -0.74 | 0.907 |
| Observations  Marginal R^2^ / Conditional R^2^ | | 3600  0.012 / 0.205 | |  |
|  |  |  |  |  |
| SE = standard error of the mean, tDCS = transcranial direct current stimulation | | | | |

**Table 3D.** Mixed effects logistic regression results predicting the probability of a higher numerical value decision in task phase 3 from tDCS condition, trial number and shock anxiety (*n* = 87).

| *Predictors* | *Odds ratio* | *SE* | *z* | *p (corrected)* |
| --- | --- | --- | --- | --- |
| Intercept | 0.95 | 0.11 | -0.47 | 0.969 |
| tDCS | 1.08 | 0.12 | 0.71 | 0.907 |
| Trial | 1.01 | 0.00 | 3.18 | **0.006** |
| Shock anxiety | 0.97 | 0.11 | -0.28 | 0.969 |
| tDCS * trial | 1.00 | 0.00 | -0.97 | 0.907 |
| tDCS * shock anxiety | 0.97 | 0.11 | -0.26 | 0.969 |
| Trial * shock anxiety | 1.00 | 0.00 | -1.23 | 0.830 |
| tDCS * trial * shock anxiety | 1.00 | 0.00 | -0.71 | 0.907 |
| Observations  Marginal R^2^ / Conditional R^2^ | | 3480  0.009 / 0.204 | |  |
|  |  |  |  |  |
| SE = standard error of the mean, tDCS = transcranial direct current stimulation | | | | |

**Table 3E.** Mixed effects logistic regression results predicting the probability of a higher numerical value decision in task phase 3 from tDCS condition, trial number and state anger (*n* = 90).

| *Predictors* | *Odds ratio* | *SE* | *z* | *p (corrected)* |
| --- | --- | --- | --- | --- |
| Intercept | 0.95 | 0.11 | -0.45 | 0.969 |
| tDCS | 1.10 | 0.12 | 0.87 | 0.907 |
| Trial | 1.01 | 0.00 | 3.37 | **0.006** |
| State anger | 1.05 | 0.14 | 0.37 | 0.969 |
| tDCS * trial | 1.00 | 0.00 | -0.74 | 0.907 |
| tDCS * state anger | 0.91 | 0.12 | -0.74 | 0.907 |
| Trial * state anger | 1.00 | 0.00 | 0.59 | 0.969 |
| tDCS * trial * state anger | 1.00 | 0.00 | 0.38 | 0.969 |
| Observations  Marginal R^2^ / Conditional R^2^ | | 3600  0.010 / 0.204 | |  |
|  |  |  |  |  |
| SE = standard error of the mean, tDCS = transcranial direct current stimulation | | | | |

**Table 3F.** Mixed effects logistic regression results predicting the probability of a higher numerical value decision in task phase 3 from tDCS condition, trial number and trait impulsivity (*n* = 90).

| *Predictors* | *Odds ratio* | *SE* | *z* | *p (corrected)* |
| --- | --- | --- | --- | --- |
| Intercept | 0.95 | 0.10 | -0.47 | 0.969 |
| tDCS | 1.10 | 0.12 | 0.88 | 0.907 |
| Trial | 1.01 | 0.00 | 3.64 | **<0.001** |
| Trait impulsivity | 1.19 | 0.13 | 1.58 | 0.490 |
| tDCS * trial | 1.00 | 0.00 | -0.72 | 0.907 |
| tDCS * trait impulsivity | 0.86 | 0.09 | -1.40 | 0.668 |
| Trial * trait impulsivity | 1.00 | 0.00 | 0.63 | 0.945 |
| tDCS * trial * trait impulsivity | 1.01 | 0.00 | 2.80 | **0.025** |
| Observations  Marginal R^2^ / Conditional R^2^ | | 3600  0.019 / 0.204 | |  |
|  |  |  |  |  |
| SE = standard error of the mean, tDCS = transcranial direct current stimulation | | | | |

**Table 4.** Descriptive statistics of the self-reported sensations of cerebellar tDCS per tDCS condition. Possible intensity scores (presented as *M* ± *SD*) range from 1 (none) to 5 (unbearable). For the onset and offset of sensations, the proportion of scores are indicated (0 = not applicable because the sensation was not perceived or data is missing, 1 = at the beginning of the stimulation, 2 = during the stimulation, 3 = towards the end of stimulation).

|  | Sham tDCS | | | Active tDCS | | |
| --- | --- | --- | --- | --- | --- | --- |
| Sensation | Intensity | Onset | Offset | Intensity | Onset | Offset |
| Itch | 1.96 ± 0.98  (*n* = 45) | 0 = 37.8 %  1 = 57.8 %  2 = 4.4 %  3 = 0 % | 0 = 37.8 %  1 = 53.3 %  2 = 4.4 %  3 = 4.4 % | 1.98 ± 1.02  (*n* = 44) | 0 = 35.6 %  1 = 51.1 %  2 = 11.1 %  3 = 2.2 % | 0 = 37.8 %  1 = 35.6 %  2 = 20 %  3 = 6.7 % |
| Pain | 1.89 ± 0.96  (*n* = 45) | 0 = 40 %  1 = 51.1 %  2 = 8.9 %  3 = 0 % | 0 = 40 %  1 = 46.7 %  2 = 11.1 %  3 = 2.2 % | 1.73 ± 0.90  (*n* = 44) | 0 = 51.1 %  1 = 42.2 %  2 = 6.7 %  3 = 0 % | 0 = 53.3 %  1 = 33.3 %  2 = 11.1 %  3 = 2.2 % |
| Burning sensation | 1.93 ± 0.89  (*n* = 45) | 0 = 33.3 %  1 = 57.8 %  2 = 6.7 %  3 = 2.2 % | 0 = 35.6 %  1 = 55.6 %  2 = 4.4 %  3 = 4.4 % | 1.86 ± 1.05  (*n* = 44) | 0 = 48.9 %  1 = 44.4 %  2 = 6.7 %  3 = 0 % | 0 = 48.9 %  1 = 37.8 %  2 = 11.1 %  3 = 2.2 % |
| Heat under electrodes | 1.60 ± 0.78  (*n* = 45) | 0 = 57.8 %  1 = 33.3 %  2 = 6.7 %  3 = 2.2 % | 0 = 57.8 %  1 = 31.1 %  2 = 8.9 %  3 = 2.2 % | 1.59 ± 0.90  (*n* = 44) | 0 = 62.2 %  1 = 31.1 %  2 = 6.7 %  3 = 0 % | 0 = 62.2 %  1 = 24.4 %  2 = 11.1 %  3 = 2.2 % |
| Iron taste | 1 ± 0 (*n* = 44) | 0 = 100 %  1 = 0 %  2 = 0 %  3 = 0 % | 0 = 100 %  1 = 0 %  2 = 0 %  3 = 0 % | 1.04 ± 0.22  (*n* = 42) | 0 = 95.6 %  1 = 0 %  2 = 0 %  3 = 4.4 % | 0 = 93.3 %  1 = 2.2 %  2 = 0 %  3 = 4.4 % |
| *Continued on the next page* | | | | | | |

| *Table 4 continued* | | | | | | |
| --- | --- | --- | --- | --- | --- | --- |
|  | Sham tDCS | | | Active tDCS | | |
| Sensation | Intensity | Onset | Offset | Intensity | Onset | Offset |
| Fatigue | 1.25 ± 0.62  (*n* = 44) | 0 = 82.2 %  1 = 2.2 %  2 = 11.1 %  3 = 4.4 % | 0 = 82.2 %  1 = 2.2 %  2 = 8.9 %  3 = 6.7 % | 1.48 ± 0.63  (*n* = 42) | 0 = 62.2 %  1 = 2.2 %  2 = 17.8 %  3 = 17.8 % | 0 = 62.2 %  1 = 2.2 %  2 = 13.3 %  3 = 22.2 % |
| Headache | 1.23 ± 0.57  (*n* = 44) | 0 = 80 %  1 = 4.4 %  2 = 11.1 %  3 = 4.4 % | 0 = 80 %  1 = 4.4 %  2 = 6.7 %  3 = 8.9 % | 1.31 ± 0.52  (*n* = 42) | 0 = 73.3 %  1 = 8.9 %  2 = 2.2 %  3 = 15.6 % | 0 = 73.3 %  1 = 4.4 %  2 = 4.4 %  3 = 17.8 % |
| Neck pain | 1.07 ± 0.45  (*n* = 44) | 0 = 97.8 %  1 = 0 %  2 = 2.2 %  3 = 0 % | 0 = 97.8 %  1 = 0 %  2 = 0 %  3 = 2.2 % | 1.07 ± 0.26  (*n* = 42) | 0 = 93.3 %  1 = 2.2 %  2 = 0 %  3 = 4.4 % | 0 = 93.3 %  1 = 0 %  2 = 2.2 %  3 = 4.4 % |
| Phosphenes | 1.02 ± 0.15  (*n* = 44) | 0 = 97.8 %  1 = 0 %  2 = 2.2 %  3 = 0 % | 0 = 97.8 %  1 = 0 %  2 = 2.2 %  3 = 0 % | 1.02 ± 0.15  (*n* = 42) | 0 = 97.8 %  1 = 2.2 %  2 = 0 %  3 = 0 % | 0 = 97.8 %  1 = 2.2 %  2 = 0 %  3 = 0 % |
| Dizziness | 1.26 ± 0.49  (*n* = 43) | 0 = 77.8 %  1 = 15.6 %  2 = 4.4 %  3 = 2.2 % | 0 = 77.8 %  1 = 13.3 %  2 = 6.7 %  3 = 2.2 % | 1.48 ± 0.63  (*n* = 42) | 0 = 60 %  1 = 22.2 %  2 = 6.7 %  3 = 11.1% | 0 = 62.2 %  1 = 20 %  2 = 6.7 %  3 = 11.1 % |
| Nausea | 1.02 ± 0.15  (*n* = 43) | 0 = 97.8 %  1 = 2.2 %  2 = 0 %  3 = 0 % | 0 = 97.8 %  1 = 2.2 %  2 = 0 %  3 = 0 % | 1.07 ± 0.26  (*n* = 44) | 0 = 93.3 %  1 = 4.4 %  2 = 2.2 %  3 = 0 % | 0 = 93.3 %  1 = 2.2 %  2 = 2.2 %  3 = 2.2 % |
| Concentration problems | 1.37 ± 0.66  (*n* = 43) | 0 = 66.7 %  1 = 11.1 %  2 = 17.8 %  3 = 4.4 % | 0 = 66.7 %  1 = 8.9 %  2 = 15.6 %  3 = 8.9 % | 1.61 ± 0.78  (*n* = 44) | 0 = 55.6 %  1 = 8.9 %  2 = 22.2 %  3 = 13.3 % | 0 = 55.6 %  1 = 2.2 %  2 = 22.2 %  3 = 20 % |
| SE = standard error of the mean, tDCS = transcranial direct current stimulation | | | | | | |

**Table 5A.** Mixed effects logistic regression results predicting the probability of a higher numerical value decision in task phase 2 from tDCS condition, trial number, shock anxiety and dizziness (*n* = 83).

| *Predictors* | *Odds ratio* | *SE* | *z* | *p (uncorrected)* |
| --- | --- | --- | --- | --- |
| Intercept | 1.54 | 0.20 | 3.38 | 0.001 |
| tDCS | 1.06 | 0.14 | 0.47 | 0.642 |
| Trial | 0.98 | 0.00 | -5.58 | **<0.001** |
| Shock anxiety | 0.94 | 0.12 | -0.47 | 0.638 |
| Dizziness | 1.01 | 0.09 | 0.12 | 0.906 |
| tDCS * trial | 1.00 | 0.00 | -0.02 | 0.984 |
| tDCS * shock anxiety | 0.81 | 0.11 | -1.57 | 0.116 |
| Trial * shock anxiety | 1.00 | 0.00 | -0.14 | 0.888 |
| tDCS * trial * shock anxiety | 1.01 | 0.00 | 2.95 | **0.003** |
| Number of observations  Marginal R^2^ / Conditional R^2^ | | 3320  0.031 / 0.169 | |  |
|  |  |  |  |  |
| SE = standard error of the mean, tDCS = transcranial direct current stimulation | | | | |

**Table 5B.** Mixed effects logistic regression results predicting the probability of a higher numerical value decision in task phase 3 from tDCS condition, trial number, trait impulsivity and dizziness (*n* = 85).

| *Predictors* | *Odds ratio* | *SE* | *z* | *p (uncorrected)* |
| --- | --- | --- | --- | --- |
| Intercept | 0.94 | 0.11 | -0.57 | 0.570 |
| tDCS | 1.15 | 0.13 | 1.20 | 0.231 |
| Trial | 1.01 | 0.00 | 3.50 | **<0.001** |
| Trait impulsivity | 1.19 | 0.14 | 1.49 | 0.137 |
| Dizziness | 0.97 | 0.10 | -0.30 | 0.762 |
| tDCS * trial | 1.00 | 0.00 | -1.33 | 0.183 |
| tDCS * trait impulsivity | 0.88 | 0.10 | -1.11 | 0.265 |
| Trial * trait impulsivity | 1.00 | 0.00 | 0.53 | 0.593 |
| tDCS * trial * trait impulsivity | 1.01 | 0.00 | 2.46 | **0.014** |
| Number of observations  Marginal R^2^ / Conditional R^2^ | | 3400  0.018 / 0.211 | |  |
|  |  |  |  |  |
| SE = standard error of the mean, tDCS = transcranial direct current stimulation | | | | |

**Table 5C.** Mixed effects logistic regression results predicting the probability of a higher numerical value decision in task phase 2 from tDCS condition, trial number, shock anxiety and fatigue (*n* = 83).

| *Predictors* | *Odds ratio* | *SE* | *z* | *p (uncorrected)* |
| --- | --- | --- | --- | --- |
| Intercept | 1.52 | 0.20 | 3.26 | 0.001 |
| tDCS | 1.04 | 0.13 | 0.34 | 0.734 |
| Trial | 0.98 | 0.00 | -5.34 | **<0.001** |
| Shock anxiety | 0.94 | 0.12 | -0.51 | 0.612 |
| Fatigue | 1.01 | 0.09 | 0.06 | 0.950 |
| tDCS * trial | 1.00 | 0.00 | 0.20 | 0.841 |
| tDCS * shock anxiety | 0.81 | 0.11 | -1.61 | 0.108 |
| Trial * shock anxiety | 1.00 | 0.00 | -0.05 | 0.957 |
| tDCS * trial * shock anxiety | 1.01 | 0.00 | 3.02 | **0.003** |
| Number of observations  Marginal R^2^ / Conditional R^2^ | | 3320  0.030 / 0.168 | |  |
|  |  |  |  |  |
| SE = standard error of the mean, tDCS = transcranial direct current stimulation | | | | |

**Table 5D.** Mixed effects logistic regression results predicting the probability of a higher numerical value decision in task phase 3 from tDCS condition, trial number, trait impulsivity and fatigue (*n* = 86).

| *Predictors* | *Odds ratio* | *SE* | *z* | *p (uncorrected)* |
| --- | --- | --- | --- | --- |
| Intercept | 0.92 | 0.11 | -0.67 | 0.501 |
| tDCS | 1.14 | 0.14 | 1.07 | 0.286 |
| Trial | 1.01 | 0.00 | 4.27 | **<0.001** |
| Trait impulsivity | 1.18 | 0.14 | 1.42 | 0.157 |
| Fatigue | 0.97 | 0.10 | -0.26 | 0.795 |
| tDCS * trial | 1.00 | 0.00 | -0.75 | 0.452 |
| tDCS * trait impulsivity | 0.87 | 0.10 | -1.14 | 0.254 |
| Trial * trait impulsivity | 1.00 | 0.00 | 0.77 | 0.439 |
| tDCS * trial * trait impulsivity | 1.01 | 0.00 | 2.91 | **0.004** |
| Number of observations  Marginal R^2^ / Conditional R^2^ | | 3440  0.023 / 0.201 | |  |
|  |  |  |  |  |
| SE = standard error of the mean, tDCS = transcranial direct current stimulation | | | | |
